# Hemosiderotic fibrohistiocytic lipomatous lesion: case report and review of the literature

**DOI:** 10.1590/S1516-31802009000300012

**Published:** 2009-10-06

**Authors:** Antônio Roberto Oliveira Ramalho, Marcella Nara Nunes, Sheila Jorge Adad, Sebastião Almeida Leitão, Adilha Misson Rua Micheletti

**Affiliations:** 1 MD. Pathology resident, Universidade Federal do Triângulo Mineiro (UFTM), Uberaba, Minas Gerais, Brazil.; 2 Medical student, Universidade Federal do Triângulo Mineiro (UFTM), Uberaba, Minas Gerais, Brazil.; 3 PhD. Associate professor, Discipline of Special Pathology, Universidade Federal do Triângulo Mineiro (UFTM), Uberaba, Minas Gerais, Brazil.; 4 MD. Adjunct professor, Discipline of Orthopedics, Universidade Federal do Triângulo Mineiro (UFTM), Uberaba, Minas Gerais, Brazil.; 5 PhD. Adjunct professor, Discipline of Special Pathology, Universidade Federal do Triângulo Mineiro (UFTM), Uberaba, Minas Gerais, Brazil.

**Keywords:** Hemosiderin, Histiocytes, Soft tissue neoplasms, Lipoma, Venous insufficiency., Hemossiderina, Histiócitos, Neoplasias de tecidos moles, Lipoma, Insuficiência venosa.

## Abstract

**CONTEXT:**

Lesions of the adipose tissue are the most common type of soft-tissue lesion among adults.

**CASE REPORT:**

We describe the case of a 33-year-old female patient with a soft-tissue lesion in her left knee that was diagnosed as a hemosiderotic fibrohistiocytic lipomatous lesion. This type of lesion, which was described for the first time in 2000, preferentially affects the ankle region of middle-aged women with a history of previous local trauma. Lesion recurrence is common, caused by incomplete resection, although there have not yet been any reports of metastases. After a review of the literature, we describe the clinical, radiological, morphological and immunohistochemical characteristics, along with their main differential diagnoses.

## RESUMO

**CONTEXTO:**

Lesões de tecido adiposo são as mais comuns de partes moles em adultos.

**RELATO DE CASO:**

Descrevemos o caso de uma paciente do sexo feminino, 33 anos, com lesão em partes moles do joelho esquerdo diagnosticada como lesão lipomatosa fibrohistiocítica hemossiderótica. Essa lesão foi descrita pela primeira vez em 2000, acometendo preferencialmente a região do tornozelo de mulheres de meia-idade com história de trauma prévio local. Recidiva da lesão é comum devido à ressecção incompleta e não há até o momento relato de metástase. Após revisão da literatura, descrevemos as características clínicas, radiológicas, morfológicas, imunoistoquímicas assim como seus principais diagnósticos diferenciais.

## INTRODUCTION

Hemosiderotic fibrohistiocytic lipomatous lesion (HFLL) is a rare benign fibrolipomatous lesion, first described as an entity in 2000 by Marshall-Taylor and Fanburg-Smith. It accounts for 0.2% of benign lipomatous lesions.^1^

HFLL is usually superficial, solitary and circumscribed but not encapsulated. It generally affects the feet and ankles (80 to 92% of the cases), although it may appear in other locations, such as cheeks and hands.^1^^,^^2^^,^^3^ The mean age of the patients is 50.6 years and 80% are women.^1^^,^^2^^,^^3^ In 88% of the cases, there is an association with trauma.^1^^,^^2^ Despite the good prognosis, recurrence occurs in 50% because of incomplete resection.^1^^,^^2^^,^^3^^,^^4^^,^^5^ Venous stasis is implicated in the pathogenesis as an excessive tissue response.^2^ Mali’s acroangiodermatitis and vascular transformation of the lymph node sinuses, two lesions related to venous stasis, are differential diagnoses.^2^

Macroscopically, HFLL is a yellowish lesion, slightly darker than lipomas, measuring between 1 and 19 cm.^1^^,^^2^^,^^3^^,^^4^^,^^5^^,^^6^^,^^7^^,^^8^ Microscopically, it consists of mature adipose tissue without atypia, associated with fusiform cells that are accompanied by inflammatory infiltrate composed of lymphocytes, plasmocytes, histiocytes and mast cells, and abundant hemosiderin pigmentation.^1^^,^^2^^,^^3^^,^^4^ Cells with slight atypia,^1^^,^^3^ floret-like cells^1^ or osteoclast-like cells^3^ can occasionally be seen. In 20% of the cases, there are small vessels with hyalinization.^1^

HFLL is positive in most cases (77-100%) for CD34, vimentin (100%) and calponin (100%), focally positive for lysozyme and KP-1 and negative for protein S-100, desmin, smooth-muscle actin, CD68, HMB-45, epithelial membrane antigen, cytokeratins and caldesmon.^1^^,^^3^

Among the differential diagnoses, various lipomatous lesions should be considered, such as adiponecrosis, fibrolipoma, fusiform cell lipoma and liposarcoma, and also fibrohistiocytic/myofibroblastic lesions such as fibromatosis, nodular fasciitis, pseudo-Kaposi’s sarcoma, fibrohistiocytoma, dermatofibrosarcoma protuberans and pleomorphic hyalinizing angiectatic tumor. The clinical-morphological-immunohistochemical association is sufficient for correct diagnosis.^1^^,^^3^^,^^4^

## CASE REPORT

Our patient was a 33-year-old white woman with pain and tumor on the posterior face of the left knee. Three years earlier, she had twisted this knee and, since then, she had been presenting pain and progressive swelling. On physical examination, there was a large-volume soft tumor accompanied by varicose veins on the posterolateral face of the left knee. The varicose veins were painful but without signs of inflammation. She also presented difficulty in flexing the left knee, with pain on touching and when walking. The patient was HIV-positive without signs of AIDS.

Ultrasound showed an expansive heterogeneous mass, laterally to the left popliteal fossa, of 8 cm in diameter. Tomography revealed a heterogeneous lesion with lacy highlighting, affecting muscle and subcutaneous tissues in the distal third of the left thigh. Magnetic resonance characterized it as lipoma.

**Figure 1.**
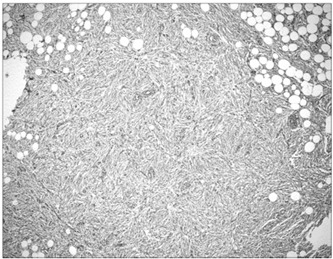
Photomicrograph of the lesion, composed of fusiform cells with a storiform growth pattern and permeated with mature adipocytes (hematoxylin-eosin, 4 x).

**Figure 2.**
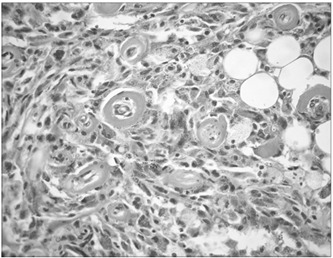
Photomicrograph of the lesion, showing histiocytes, mature adipocytes and small blood vessels with hyalinized walls (hematoxylin eosin, 20 x).

The patient underwent surgery with an incision measuring 1.5 cm on the posterior face of the knee. The lesion was excised with free margins. The material consisted of soft friable brownish-yellowish fragments that together measured 10 x 8 x 6 cm and weighed 80 g. Under the microscope, proliferation of fusiform cells without atypia was observed, with mature adipocytes. The specimen was permeated with abundant hemosiderin pigment, along with small vessels with hyalinized walls (Figures 1 and 2). At the center, there was an old hemorrhage. There was no invasion of vessels and nerves. The margins were difficult to assess because the material was fragmented. Immunohistochemistry was positive for vimentin and CD34, and negative for cytokeratin, desmin, smooth-muscle actin, HHF-35, protein S-100 and CD 99.

Fifteen months later, the patient presented with a swelling adjacent to the previous surgical scar measuring 2 x 1.5 x 0.5 cm. On re-excision, the lesion had the same features, except for more ganglionic and multinucleated cells. There were no free margins. Six months after the second excision, the patient continued to show no signs of recurrence or metastasis.

## DISCUSSION

We only found 29 cases of HFLL in PubMed^1^^,^^2^^,^^3^^,^^4^^,^^5^^,^^6^^,^^7^^,^^8^ (Table 1). Our case had some unusual characteristics and some interesting associated factors that may have been implicated in the pathogenesis, which is still a matter for discussion.

Among the 29 cases described, 22 (75.86%) were women, of mean age 50 years, and 25 cases (86.2%) were presented in the ankle. Except for one case in a child and a 32-year-old patient described by Browne and Fletcher,^3^ our patient was younger than the mean age in the literature. Moreover, our lesion is the first described in the knee. Among the others, 25 cases affected ankles, two affected the hands, one occurred in the cheek and one occurred in the thigh.^1^^,^^2^^,^^3^^,^^4^^,^^5^^,^^6^^,^^7^^,^^8^

One unusual morphological characteristic seen in our case was the hyalinization of the walls of small blood vessels, which has been seen in only 27.5% of the cases (eight cases) in other series. ^1^^,^^2^^,^^3^^,^^4^

The idea that this lesion may be associated with venous stasis was first broached by Marshall-Taylor and Fanburg-Smith in 2000, while discussing the differential diagnosis with Mali’s acroangiodermatitis. However, the idea was dismissed, given that none of the ten patients in their series presented vascular insufficiency.^1^ Nevertheless, this likely association was subsequently advocated by Kazakov et al, in relation to two patients who presented this lesion and chronic venous insufficiency.^2^ Thus, HFLL would represent an excessive tissue response to venous stasis, since raised pressure in the veins and capillaries, oxygen saturation and edema would stimulate the proliferation of the elements seen in this lesion.^2^^,^^9^ Kazakov et al. and Michal and Kazakov also showed that transformation of the lymph node sinus was another condition with morphology similar to HFLL and was also associated with venous stasis caused by occlusion of the efferent lymphatic vessels and/or veins.^2^^,^^9^

The patient in our case presented varicose veins in the affected leg and this makes us believe that venous stasis may really play an important role in the pathogenesis of this lesion.

At present, there are two major points for debate regarding HFLL: whether its nature is reactive or neoplastic; and whether it is or is not the precursor of pleomorphic hyalinizing angiectatic tumors, as advocated by Folpe and Weiss.^10^ Just like Marshall-Taylor and Fanburg-Smith and Kazakov et al., and Michael and Kazakov^1^^,^^2^^,^^9^ we believe that HFLL is reactive, bearing in mind its superficial location, frequent association with previous trauma (11 cases, i.e. 39.3%, including ours), lack of capsule, morphology of varied cells and accumulation of hemosiderin. In our case, we also observed a hematoma at the center of the lesion, thus confirming the history of previous trauma and the possibility of a reactive lesion.

**Table 1.** Database search strategies for hemosiderotic fibrohistiocytic lipomatous lesion

Database	Search strategy	Results
PubMed	(“Histiocytoma, Malignant Fibrous”[MeSH]) OR (Fibrous Histiocytoma, Malignant) Or (Fibrous Histiocytomas, Malignant) Or (Histiocytomas, Malignant Fibrous) Or (Malignant Fibrous Histiocytomas) Or (Malignant Fibrous Histiocytoma) Or (Fibrohistiocytic) AND (“Neoplasms, Adipose Tissue”[MeSH]) OR (Adipose Tissue Neoplasms) OR (Adipose Tissue Neoplasm) OR (Neoplasm, Adipose Tissue) OR (LIPOMATOSIS) OR (LIPOMATOSES) OR (“Neoplasms, Fibrous Tissue”[MeSH]) OR (Lipomatous Lesion) or (Lipomatous Tumor) AND (haemosiderotic OR hemosiderotic OR hemosideroses OR hemosiderosis)	3 original articles 1 letter to editor 1 updating article 2 case series 3 case reports

## CONCLUSION

Hemosiderotic fibrohistiocytic lipomatous lesion is a recently described rare entity that usually affects middle-aged women’s feet and ankles. Complete resection is necessary in order to avoid local recurrence.

There is still much discussion regarding its reactive or neoplastic nature. This has generated controversy, and studies of greater extent are therefore needed to reach a definitive conclusion.
